# Phage Therapy for Sustainable Sea Cucumber Aquaculture

**DOI:** 10.3390/life16060989

**Published:** 2026-06-11

**Authors:** Wan Zhang, Xiaowen Sun, Bilal Murtaza, Xiaoyu Li, Lili Wang, Yongping Xu

**Affiliations:** MOE Key Laboratory of Bio-Intelligent Manufacturing, School of Bioengineering, Dalian University of Technology, Dalian 116024, Chinabilalmurtaza50@gmail.com (B.M.);

**Keywords:** phage application, environmental safety, disease outbreaks, aquaculture health management, phage cocktails, biocontrol strategies

## Abstract

Phage therapy is increasingly recognized as a promising alternative to antibiotics for controlling bacterial diseases in aquaculture. This review focuses specifically on sea cucumber farming, with emphasis on phage application methods, therapeutic performance, and current limitations in translating laboratory results into field use. Available studies show that phage-based treatments can improve the survival of *Apostichopus japonicus* challenged with *Vibrio* spp., especially when delivered through feed or formulated as phage cocktails. However, practical application is still constrained by host-range specificity, phage resistance, environmental stability, delivery efficiency, and regulatory barriers. By summarizing recent evidence and identifying research gaps, this review highlights the potential of phage therapy as a sustainable disease management strategy for sea cucumber aquaculture.

## 1. Introduction

Aquaculture has emerged as a critical sector in global food production, supplying nearly half of the world’s seafood demand, with China leading as the largest producer [1]. Among aquaculture species, sea cucumbers, particularly *Apostichopus japonicus*, have gained substantial economic importance due to their high nutritional and medicinal value [2,3]. Sea cucumber aquaculture continues to expand, with China reporting 326,000 tons of production in 2024, an increase of 34,000 tons over 2023, and market reports suggesting that China accounts for over 60% of global sea cucumber consumption (China Fishery Statistics Yearbook, 2024 [4]). However, the intensification of sea cucumber farming has exacerbated disease outbreaks, predominantly bacterial infections caused by *Vibrio* species and other opportunistic pathogens, leading to significant mortality and economic losses [5]. The prevalent use of antibiotics to manage these diseases has resulted in environmental contamination, antibiotic residues in aquatic products, and the alarming rise of antibiotic-resistant bacteria, posing serious threats to both aquaculture sustainability and public health [6].

Phage therapy research in aquaculture has expanded rapidly, but most studies remain pathogen-specific and laboratory-based. In sea cucumber farming, the evidence is promising but still fragmented, with major gaps in field validation, formulation stability, and ecological safety. In this context, bacteriophage therapy, utilizing viruses that specifically infect and lyse bacteria, has re-emerged as a promising and eco-friendly alternative for disease control in aquaculture [7]. Phages offer remarkable advantages, including high specificity to target pathogens without harming beneficial microbiota, self-replication in the presence of hosts, biodegradability, and minimal environmental impact [8]. The adaptability of phages to evolve alongside bacterial hosts makes them potent tools against antibiotic-resistant bacteria, aligning with global efforts to reduce antibiotic use in food production systems [9]. Recent advances in phage genomics, molecular biology, and biotechnology have enhanced our understanding of phage diversity, infection mechanisms, and safety, enabling the development of optimized phage cocktails tailored to specific pathogens such as *Vibrio harveyi* and *Vibrio parahaemolyticus* in aquaculture environments [10,11]. Although phage therapy has been reviewed in aquaculture, few reviews have focused specifically on sea cucumber disease management. In particular, the practical application routes of phages in *Apostichopus japonicus* farming and the limitations affecting their field use have not been discussed in sufficient detail. Therefore, this review addresses this gap by summarizing current evidence on phage therapy in sea cucumber aquaculture, with emphasis on application methods, therapeutic potential, and remaining challenges for practical implementation. This review aims to synthesize current evidence on bacteriophage therapy for sea cucumber (*Apostichopus japonicus*) aquaculture. Specifically, we address: (1) the major bacterial pathogens responsible for skin ulceration and the immune context of the host; (2) practical phage application methods and their demonstrated therapeutic performance; and (3) the principal challenges, including host-range specificity, phage resistance, environmental stability, and regulatory barriers that currently limit field implementation.

## 2. Status of Sea Cucumber and Sea Cucumber Farming

The sea cucumber (*Apostichopus japonicus*), sometimes referred to as the Japanese sea cucumber, belongs to the family *Stichopodidae* and genus *Apostichopus*. It is predominantly found along China’s coastal waters, especially around the Shandong Jiaodong Peninsula and the Liaoning coast, with Dalian and Yantai noted for producing sea cucumbers of superior quality. Of the more than 20 edible sea cucumber species documented in China, *Apostichopus japonicus* is distinguished by its high nutritional value, attributable to its rich protein content and diverse acidic mucopolysaccharides, which contribute to human metabolic regulation and immune system enhancement [12] (Table 1).

This has catalyzed rapid growth in China’s sea cucumber aquaculture over the past decade, making it the marine species with the highest individual species output value within the national aquaculture industry [13]. Sea cucumber farming has become an important aquaculture sector because it helps relieve pressure on wild stocks while meeting strong market demand in Asia, especially China. In China alone, sea cucumber production reached about 326,000 tons in 2024, and earlier reports noted that hatchery-based cultivation of *Apostichopus japonicus* could produce about 6 billion juveniles per year, showing the scale already achieved by the industry [14,15]. This expansion has been propelled by a transition from extensive to intensive and large-scale farming methods. However, intensification has also led to frequent bacterial disease outbreaks, with rotten skin syndrome being the most damaging. These disease outbreaks have caused substantial economic losses and triggered extensive use and often misuse of antibiotics and chemical treatments. Such practices have contributed to the rise of antibiotic-resistant bacterial strains, raising significant concerns for public health and the sustainability of aquaculture practices [16].

**Table 1:** Updated Sea Cucumber Species in China.

Species of Sea Cucumber	Reproductive Type	Spawning Period	Oocyte Diameter (µm)	References
*Apostichopus japonicus*	Gonochoric	June to August	120	[17]
*Holothuria scabra*	Gonochoric	April to July	150–200	[18]
*Stichopus hermanii*	Gonochoric	April to September	200	[19]
*Actinopyga lecanora*	Fission	March to July	324	[20]
*Thelenota ananas*	Gonochoric	June to August	200	[21]

**Figure 1:**
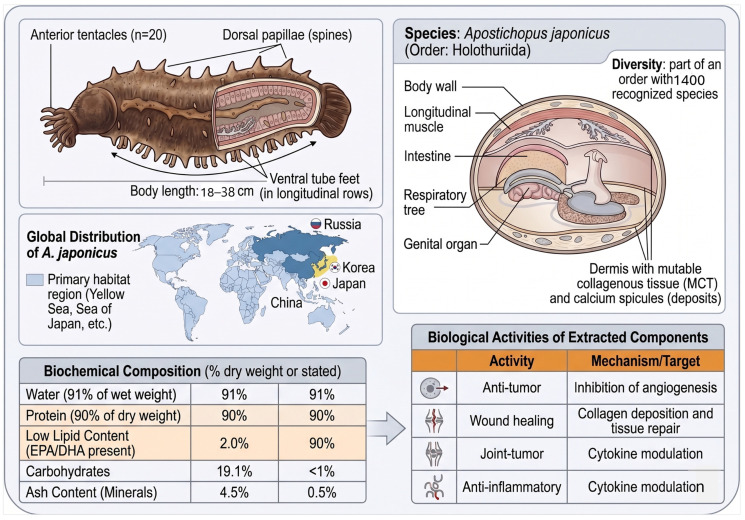
External morphology and anatomical overview of *Apostichopus japonicus*, with emphasis on tissue compartments (body wall, coelomic cavity, gut) relevant to bacterial pathogen entry and phage delivery. The inset highlights the high protein-to-lipid composition that characterizes the species’ commercial and nutritional value [25,26,27]. (Bio render Version 04).

Nutritionally, sea cucumbers have been used as a traditional food and medicinal resource in several cultures. They contain proteins, essential trace elements, vitamins, and fatty acids of nutritional interest, and their composition varies depending on species, processing method, and environmental conditions. Live sea cucumbers contain a high water content, whereas dried products are enriched in protein and contain relatively low total fat. Bacterial diseases such as Vibrio-associated skin ulceration can cause substantial losses in sea cucumber culture. Phage therapy has been proposed as a pathogen-specific approach for controlling bacterial infections in aquaculture [22,23,24] (Figure 1).

## 3. Vibrio Disease and Sea Cucumber Skin Rot Syndrome

Sea cucumber skin rot syndrome is one of the most serious bacterial diseases affecting *Apostichopus japonicus* farming, with *Vibrio* species identified among the major causative agents. Outbreaks usually begin with abnormal behavior and tissue damage and may progress rapidly to body-wall ulceration, autolysis, and death, causing substantial economic loss in seedling and grow-out systems [28,29]. Because the disease is highly infectious and often recurs in intensive farming environments, effective control strategies are urgently needed. In this context, phage therapy is particularly relevant because it offers targeted killing of pathogenic bacteria without adding chemical residues to the culture environment [30,31,32]. Numerous *Vibrio* spp. also affect fish and crustaceans (*V. anguillarum*, *V. harveyi*), but here we focus on strains implicated in sea cucumber skin ulceration [33,34] (Table 2).

Vibrio-associated disease outbreaks in sea cucumber aquaculture are influenced by multiple environmental factors, including water temperature, stocking density, and regional conditions. While *Vibrio* spp. are capable of persisting across a wide range of conditions, outbreak frequency and severity tend to increase during warmer months and in high-density culture systems, consistent with the thermal ecology of the dominant pathogens [35,36,37]. Diseases caused by Vibrio include eye rot, gill rot, tail rot, shell ulcer disease, muscle leukoplakia, yellow gill disease, larval fluorescent disease, septicemia, and skin rot [38,39,40,41]. Wang analyzed the pathogens and infection sources of these outbreaks in Shandong and classified the condition as “sea cucumber skin rot syndrome. The etiology of sea cucumber skin rot syndrome involves a clear hierarchy of infection. *Vibrio splendidus* and *Vibrio alginolyticus* are consistently identified as primary causative agents, initiating tissue damage through toxin production and enzymatic degradation of the body wall. Secondary infections by molds, parasites, and opportunistic bacteria frequently compound the initial lesions, particularly under high stocking densities and suboptimal water quality conditions. Regional variation is also evident, *Vibrio splendidus* predominates in Shandong and Liaoning, while *Aeromonas salmonicida* and *Pseudoalteromonas* spp. are more commonly reported in Fujian Province (Table 3) [42,43].

**Table 2:** Pathogenic *Vibrio* of aquatic animals.

Pathogenic Bacteria	Infected Animals	Popular Areas	References
*Vibrio anguillarum*	Prawns, fish, Sea cucumber	Europe, Asia	[44]
*Vibrio vulnificus*	Eel, prawns, fish	Spain, Asia	[45]
*Vibrio fluvialis*	Barramundi, carp, flounder	China	[46]
*Vibrio harveyi*	Sea bass, yellow croaker	Asia	[47]
*Vibrio alginolyticus*	Prawns, marine fish, and clams	Europe, Asia	[48]
*Vibrio parahaemolyticus*	Prawns, marine fish, and clams	Europe, Asia	[49]
*Vibrio mimicus*	Sea bream	Japan, China	[50]
*Vibrio carchariae*	Sea fish	Europe, Asia	[51]
*Vibrio damsela*	Marine fish, shrimp	Europe, Asia	[52]
*Vibrio ordalii*	Sea fish	Europe, Asia	[53]
*Vibrio salmonicida*	Salmon	UK, Norway	[54]
*Vibrio ichthyoenteri*	Flounder	Japan	[55]
*Vibrio cholerae*	Ayu	Japan	[56,57,58]
*Vibrio splendidus*	Sea cucumber, oyster	China, France	[59,60,61]

**Table 3:** Major pathogens of skin ulceration syndrome on sea cucumber.

Pathogens	Main Incidence Areas	References
*Vibrio splendidus*	Shandong Province, Liaoning Province, Fujian Province	[62,63,64]
*Vibrio cyclitrophicus*	Liaoning Province	[65,66,67,68]
*Vibrio alginolyticus*	Shandong Province	[69]
*Vibrio harveyi*	Liaoning Province	[70,71]
*Vibrio parahaemolyticus*	Liaoning Province	[72]
*Aeromonas salmonicida*	Shandong Province	[73]
*Pseudoalteromonas*	Shandong Province, Fujian Province	[74]
*Pseudomonas*	Liaoning Province, Shandong Province	[75]
*Shewanella smarisflavi*	Liaoning Province	[76]

## 4. Nonspecific Immunity of Sea Cucumbers

The cellular immune response in echinoderms is mediated by various free-floating coelomocytes capable of recognizing and neutralizing pathogens through phagocytosis, encapsulation, coagulation, cytotoxicity, and wound healing [77,78]. Meanwhile, humoral immunity involves secreted molecules such as lectins, agglutinins, perforins, complement proteins, and cytokines found in bodily fluids that support pathogen defense [79,80]. Sea cucumbers rely mainly on nonspecific immune defenses, including coelomocytes, phagocytosis, encapsulation, and humoral factors, to resist microbial invasion. These innate responses are especially important in marine environments where exposure to pathogens is continuous. Since phage therapy reduces bacterial load at the infection site, it may complement these natural defenses rather than replace them, thereby supporting host survival and recovery under disease pressure [81]. These immune features are conserved in sea cucumbers, whose defenses include body wall barriers, cellular immune responses, and humoral factors. The body wall functions as the first defense line, enabling sea cucumbers to thrive in marine environments rich in bacteria, fungi, and viruses. Studies have shown that extracts from sea cucumber body walls exhibit antibacterial activity against both Gram-positive and Gram-negative bacteria, with efficacy increasing alongside extract concentration [82,83]. Similar antibacterial substances resistant to proteinase K and heat, with hemolytic activity, have been identified in *Cucumaria frondosa* and are also present in the respiratory tree and gastrointestinal tissues [84].

Within the coelomic cavity, coelomic fluid houses coelomocytes crucial for immune defense. In *Apostichopus japonicus*, six major coelomocyte types have been characterized: progenitor cells, amoebocytes, vibratile cells, crystal cells, morula cells, and vacuolated cells, with progenitor cells being the most abundant, followed by amoebocytes [85,86]. Despite residing in pathogen-dense environments, the coelomic fluid remains nearly sterile, likely due to the efficient pathogen clearance by coelomocytes. Phagocytosis and encapsulation are central defense strategies, primarily mediated by amoeboid phagocytes that exist in petal-shaped forms for engulfing particles and filamentous forms involved in coagulation and wound repair [3]. These cells contain lysosomal enzymes such as acid phosphatase, alkaline phosphatase, aminopeptidase, and lipase, which facilitate enzymatic degradation of foreign substances [87,88]. These innate immune features have direct implications for phage therapy. The presence of phagocytic coelomocytes and humoral antimicrobial factors means that introduced phage particles may be subject to non-specific clearance alongside bacterial targets. Studies in other invertebrate models suggest that the host immune environment can reduce phage persistence at infection sites, potentially requiring higher phage doses or repeated administration to maintain therapeutic titers. Conversely, phage-mediated reduction in bacterial load may reduce the immunological burden on coelomocytes, indirectly supporting host survival and recovery. Understanding these host phage pathogen interactions in *A. japonicus* represents an important direction for future mechanistic research [89,90,91,92].

## 5. Milestones in Phage Research

**Figure 2:**
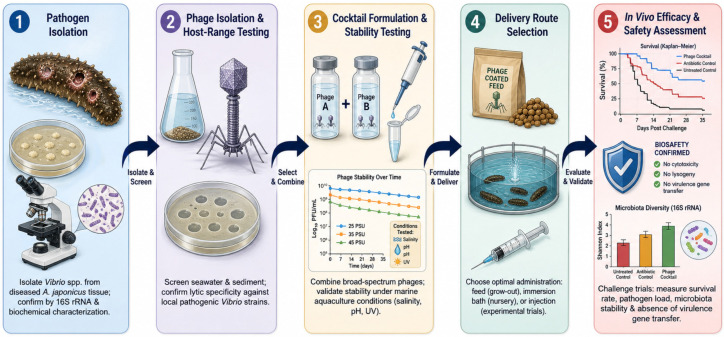
Workflow for phage therapy application in *Apostichopus japonicus* aquaculture (Bio render).

Bacteriophage research originated in 1917 when French-Canadian microbiologist Félix d’Hérelle identified a bacterial-destroying agent capable of curing dysentery patients, coining the term “phage” from the Greek phagein, meaning “to eat” [93] (Figure 2). Together with Frederick Twort, d’Hérelle established bacteriophage therapy as a recognized approach against infections, including avian typhoid fever, bovine hemorrhagic septicemia, bacillary dysentery, and bubonic plague [94,95,96,97], demonstrating early on that phages could be applied across both human and animal disease contexts, a principle that remains central to their current evaluation in aquaculture systems.

Renewed institutional momentum emerged in 2015, when the U.S. National Institute of Allergy and Infectious Diseases formally endorsed phage therapy as a strategy for combating antibiotic resistance, and the European Union funded the Phagoburn project with $5.2 million to conduct clinical trials targeting *E. coli* and *P. aeruginosa* in burn wound patients across France, Belgium, and the Netherlands [98,99]. In 2018, the U.S. FDA granted emergency use authorization for compassionate phage therapy in antibiotic-resistant cases [100,101,102,103,104,105], and the European Medicines Agency began developing regulatory guidelines for phage-based medicinal products. In April 2024, the European Pharmacopoeia adopted a dedicated chapter establishing quality control and manufacturing standards for phage-based products, while the FDA has advanced discussions on standardized approval pathways for phage cocktails and genetically modified phages [106,107]. While these regulatory advances are primarily directed at human medicine, they establish critical precedents, including phage safety profiling, manufacturing consistency requirements, and environmental release assessment frameworks that are directly applicable to veterinary and aquaculture phage product development. In aquaculture specifically, regulatory approval for phage-based treatments remains at an early stage in most jurisdictions, and coordinated alignment between food safety agencies and aquaculture regulators will be necessary before phage preparations can be licensed for commercial farm use, including sea cucumber aquaculture. An effective phage cocktail, whether for clinical or aquaculture application, must combine complementary host ranges, strong lytic activity, genetic safety, and formulation stability while minimizing antagonism among component phages [108,109].

## 6. Phage Classification and Biological Characteristics

Phage taxonomy has shifted from the classical morphology-based families *Myoviridae*, *Podoviridae*, and *Siphoviridae* to the updated ICTV framework, which uses refined higher-level classifications; therefore, the Supplementary Table is presented here for historical context and comparative reference. In 1966, the International Committee for the Nomenclature of Viruses (ICNV) and the Subcommittee on Bacterial Viruses were established to oversee virus taxonomy [110,111]. The ICTV’s 10th Report, published in 2017, provided a comprehensive update on virus taxonomy, incorporating molecular and morphological data [17]. Despite advances in molecular biology and genomics, virus classification still fundamentally relies on characteristics such as virion morphology, nucleic acid type, and host range. Viruses are primarily grouped based on their genome type into single-stranded DNA (ssDNA), double-stranded DNA (dsDNA), single-stranded RNA (ssRNA), and double-stranded RNA (dsRNA) viruses. Taxonomic hierarchy is structured into five levels: Order, Family, Subfamily, Genus, and Species [112]. Currently, bacteriophages are classified into over 20 families, with approximately 96% belonging to the order *Caudovirales* [113]. Historically, *Caudovirales* were divided into three families based on tail morphology, *Podoviridae* (short tails, ~14%), *Siphoviridae* (long non-contractile tails, ~61%), and *Myoviridae* (contractile tails, ~25%) [114,115]. However, recent ICTV reports have refined this classification, reflecting genomic and proteomic insights. This approach addresses the vast genetic diversity unveiled by viral metagenomics. Table S1 summarizes representative classification statistics for each genus within Caudovirales according to the ICTV Master Species List 2023 v1.0 (ICTV, 2023 [116]), illustrating the dynamic evolution of phage taxonomy and the necessity for continual updates as new viral genomes emerge.

**Figure 3:**
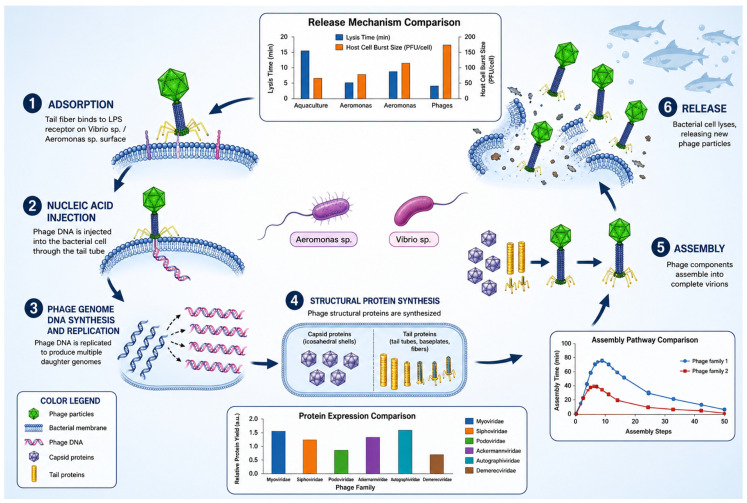
Life cycle of lytic phages.

The protein coat, or capsid, typically consists of a head and tail structure (Figure 3) [117,118,119,120]. The genetic material of phages can be either DNA or RNA, and their genomes may be linear or circular [19]. While most phage capsids contain only nucleic acids and proteins, a small subset also incorporates lipids or sugars within their heads [121,122]. Importantly, bacteriophages lack the cellular machinery necessary for self-replication and metabolism; thus, they depend entirely on the host bacterial cell’s genetic translation system for replication and propagation [123,124].

Phages are classified into two main types based on their interaction with the host: lytic phages and lysogenic (temperate) phages [125]. Lytic phages infect bacterial cells, replicate rapidly, and cause host cell lysis to release progeny phages. Lysogenic phages, in contrast, integrate their genome into the host bacterial chromosome, existing as a prophage that replicates passively with the host cell [126,127]. Figure 3 depicts the lytic phage life cycle, outlining these stages and the dynamic host-phage interaction.

### 6.1. Adsorption and Penetration

Adsorption is the initial and essential step in the bacteriophage infection cycle, where phage particles specifically bind to receptors on the bacterial host cell surface. This binding is mediated by tail-specific proteins that recognize unique molecular structures such as lipopolysaccharides, teichoic acids, or membrane proteins [18,128,129,130]. The specificity of this interaction determines the phage’s host range, as infection occurs only in bacteria presenting compatible receptors [131].

Following adsorption, tailed bacteriophages of the order *Caudovirales* inject their genetic material into the bacterial host through a coordinated mechanism involving enzymatic degradation and mechanical force [132,133]. Initially, phage-associated enzymes like terminal lysozymes locally degrade the bacterial peptidoglycan layer, creating an entry point through the rigid cell wall [134]. The contractile tail sheath then contracts, driving the tail tube through the compromised envelope and injecting the phage genome into the host cytoplasm, functioning like a molecular syringe powered by stored tail energy [135]. This process is tightly regulated to ensure precise genome delivery. Beyond enzymatic and mechanical actions, some phages hijack host membrane proteins or efflux channels to facilitate genome translocation across the inner membrane, utilizing bacterial transport systems to breach this barrier [136].

### 6.2. DNA Synthesis and Morphogenesis

Following adsorption, the host RNA polymerase (RNAP) recognizes strong early phage promoters and initiates transcription of small precursor genes [137]. These early gene products redirect host transcriptional machinery to prioritize phage gene expression by inhibiting host promoter activity and inactivating restriction endonucleases, thus protecting phage DNA and ensuring efficient phage mRNA synthesis [20,138]. Subsequently, RNAP transcribes middle and late phage genes encoding structural proteins essential for virion assembly [139]. Phage DNA, often replicated as concatemers, is packaged into the procapsid through a portal complex powered by an ATP-driven motor [140,141]. Notably, the T4 phage packaging machine exhibits flexibility by translocating DNA into both immature proheads and mature capsids [142,143]. Once packaging is complete, tail proteins assemble onto the filled head to form mature virions. Tail assembly starts with tail-pin proteins, followed by neck proteins that connect the tail and head, sealing the virion. This highly regulated temporal and spatial assembly ensures production of infectious progeny [144,145]. The timing of host cell lysis is crucial for maximizing phage fitness. If lysis occurs too rapidly, only a limited number of phages may be produced and released, reducing the overall yield of infectious particles. Conversely, if lysis is excessively delayed, opportunities for subsequent infections diminish, and the production of secondary phages is compromised, potentially limiting the spread of the phage population [100,146,147].

## 7. Phage Application Methods in Sea Cucumber Aquaculture

Phage application in sea cucumber aquaculture has been demonstrated through several practical delivery routes, including oral administration, feed supplementation, immersion, and coelomic injection. In juvenile *Apostichopus japonicus*, phage-containing diets significantly improved survival after *Vibrio splendidus* challenge, and phage cocktails performed better than single-phage treatments, showing that feed-based delivery can be an effective and non-stressful approach for disease control in sea cucumber farming [148,149,150]. Similar results were reported for phage cocktails against *Vibrio parahaemolyticus*, where incorporation into feed with a suitable protectant improved therapeutic performance without negatively affecting growth or feed utilization [151]. More recent aquaculture reviews emphasize that the delivery route should be matched to the pathogen distribution, production stage, and farming environment, with oral and immersion routes often being the most practical for large-scale use, while injection remains more suitable for controlled experimental settings. Together, these studies indicate that phage therapy is most promising when formulated as a targeted, stable, and farm-specific intervention rather than as a single universal treatment strategy [152,153,154].

## 8. Phage Therapy in Sea Cucumber Disease Management

Phage application in sea cucumber aquaculture can be achieved through several practical routes, including immersion, feed-based delivery, and phage cocktails. In juvenile *Apostichopus japonicus*, feed-based phage administration has shown clear protective effects against *Vibrio* infection, while cocktail formulations are often more effective than single-phage treatments because they broaden host coverage and reduce the risk of resistance. These delivery approaches are most useful when matched to the pathogen, farming stage, and environmental conditions, making formulation and timing important for successful application [155,156].

Phage therapy has shown promising efficacy against Vibrio-mediated diseases in sea cucumbers (*Apostichopus japonicus*), particularly skin ulceration syndrome. Phage PVA1, isolated against *Vibrio alginolyticus* (a dominant pathogen in Shandong/Liaoning outbreaks), demonstrates strong lytic activity, achieving 90% bacterial clearance (MOI 10, 24 h) and improving juvenile survival 75–85% in immersion challenges [69]. Li Zhen’s dissertation characterized phage cocktails (PVS-1, PVS-2, PVS-3) targeting *V. splendidus*, reducing mortality from 80% to 20% in infected juveniles via coelomocyte activation (lysozyme ↑2.3-fold, SOD ↑1.8-fold; 48 h post-challenge [157]. Cao, who isolated vB_VneM_NB-1 against *V. nereis*, a coelomocyte apoptosis inducer, demonstrating 81% survival uplift (feed immersion, juveniles) and inhibition of caspase-3 activation (↓64%; burst size 215 PFU/cell, eclipse 25 min) [60].

## 9. Phage Control of Major Aquaculture Pathogens

Phage therapy in aquaculture has therapeutic potential, but its effectiveness depends on multiple interacting factors. The therapeutic value of phage therapy in aquaculture is shaped by an interaction among phage host range, bacterial resistance evolution, environmental stability, and formulation design rather than by lytic activity alone. Because temperate phages may contribute to lysogenic conversion or transduction, strictly lytic phages are generally preferred for therapeutic use in aquaculture. This preference reduces the risk of transferring virulence or antimicrobial-resistance genes to bacterial populations.

### 9.1. Analysis of the Necessity of Antibiotic Substitution in Aquaculture

Over the past decade, China’s aquaculture industry has experienced remarkable growth, becoming a vital part of the national economy. As of 2014, China’s aquaculture output accounted for more than 70% of the world’s total production, making it the only country where aquaculture output surpasses that of wild capture fisheries [158,159]. According to the Food and Agriculture Organization of the United Nations (FAO), declining yields in offshore fisheries due to overfishing were projected to make aquaculture supply half of global seafood demand by 2020 [1]. China remains the world’s largest producer of aquatic products, with total output reaching approximately 71.16 million tons in 2023, showing a steady increase from 68.66 million tons in 2022 [160].

According to the 2017 China Fisheries Yearbook, in 2016, China’s total aquatic product output was 69.01 million tons, with aquaculture contributing 51.42 million tons, representing 74.5% of total production. Marine products accounted for 34.9 million tons (50.6% of total output), of which marine aquaculture contributed 19.63 million tons, or 56.3% of marine production, with a production value of 314 billion yuan, a 4.67% increase year-on-year (China Fisheries Yearbook, 2017 [161]). The rapid expansion of aquaculture, especially marine aquaculture, is largely attributed to intensive, large-scale farming models. Antibiotics have played a crucial role in supporting this growth. In aquaculture production, antibiotics are used not only to prevent and control bacterial disease outbreaks but also to promote growth and improve feed efficiency [162]. However, the high-density nature of aquaculture systems increases the risk of widespread bacterial infections, which further elevates antibiotic usage.

While antibiotics have been instrumental in disease control, their overuse and misuse have led to significant problems. Many aquaculture operators lack adequate knowledge regarding proper dosages and usage standards, and insufficient regulatory oversight has allowed antibiotics to be abused as a “panacea” for disease prevention. This has resulted in environmental contamination, the emergence of antibiotic-resistant bacteria, and residues in aquatic products, posing serious food safety and public health risks [163]. Consequently, numerous Chinese aquatic product exports have been rejected by foreign customs due to antibiotic residues, causing substantial economic losses and damaging China’s international reputation.

The broad-spectrum use of antibiotics disrupts aquatic ecosystems and alters the normal gut microbiota of farmed species, ultimately reducing aquaculture efficiency. In July 2016, a CCTV investigation exposed antibiotic abuse at a sea cucumber farm in Dalian, resulting in the near extinction of offshore species, severe ecological damage, eutrophication, and nitrogen-phosphorus imbalances, highlighting the urgent need for sustainable aquaculture practices. In response, Chinese authorities have tightened regulations on antibiotic use, banning several substances, including ciprofloxacin, enrofloxacin, nitrofurans like furazolidone, and feed additives such as erythromycin and chloramphenicol [164]. These stricter policies and growing concerns over residues have intensified the demand for alternative antibacterial strategies. Historically, during the Cold War, Western antibiotic embargoes prompted the former Soviet Union to continue employing phage therapy against bacterial diseases. With the current global rise in antibiotic resistance and superbugs, phage therapy has regained international attention, with many countries advancing its application to control bacterial infections and achieving promising outcomes [165]. Consequently, implementing phage therapy in aquaculture, particularly sea cucumber farming, is of great practical importance for sustainable disease management and environmental conservation.

### 9.2. Phages for the Prevention and Control of Aquaculture Pathogens

Phage therapy involves using bacteriophage viruses that specifically infect and lyse bacteria to treat bacterial infections in humans and animals. It is a biological control method that leverages the natural antibacterial properties of phages [100]. In aquaculture, phage application to prevent and manage bacterial diseases offers several significant advantages. First, bacteriophages demonstrate high efficiency and exceptional host specificity, allowing them to selectively target pathogenic bacteria while sparing beneficial microbial communities. This specificity helps preserve the ecological balance and microecological stability within aquaculture environments [166]. Second, their capacity for self-replication within target bacteria means only a small initial phage dose is needed to achieve effective pathogen control, enhancing cost-effectiveness and efficiency [167]. Third, phages are naturally abundant, and phage isolates specific to most aquaculture pathogens can be readily obtained from water, sediments, and infected hosts [168]. Fourth, phage isolation and purification are relatively rapid, and their ease of storage and application facilitate practical use in aquatic systems [169]. Lastly, the small size and well-characterized genomes of phages allow thorough sequencing and toxicity testing, minimizing horizontal gene transfer risks and ensuring the safety of phage preparations [100]. Successful phage applications in aquaculture worldwide are summarized in Table 4, highlighting their potential against major pathogens.

### 9.3. Phage Control of Lactococcus garvieae

*Lactococcus garvieae* is a Gram-positive bacterium from the genus *Lactococcus* and a major pathogen in aquaculture, causing lactococcosis in species like yellowtail amberjack (*Seriola quinqueradiata*), rainbow trout (Oncorhynchus mykiss), gilthead sea bream, and European seabass [185,186]. The disease manifests through symptoms such as enteritis, abdominal hemorrhage, anorexia, melanosis, exophthalmia, and swim bladder ataxia, often resulting in mortality rates exceeding 50%, especially at elevated water temperatures [186,187]. As a conditional pathogen commonly found in aquaculture environments, *L. garvieae* outbreaks occur under stress or immunosuppression [188].

Researchers have isolated multiple lytic bacteriophages targeting *L. garvieae*, primarily from the PLgY and PLgW series [189]. Screening of 111 *L. garvieae* strains identified 14 phage types, with about 90% showing susceptibility to phages PLgW-1 and PLgW-3. These phages demonstrated short incubation periods (~1 h) and high titers (up to 10^10^ PFU/mL) within 5 h post-infection. Therapeutic trials revealed 100% survival in infected fish treated via intraperitoneal phage injection versus 10% in controls, while oral administration also significantly reduced mortality, with phages persisting in intestines and spleen up to 48 h at titers of 10^6^ PFU/g [190]. These findings highlight the efficacy of *L. garvieae*-specific phages and inform optimal treatment strategies.

In addition to phage therapy, *Lactobacillus gasseri* is a probiotic bacterium that has been studied for beneficial effects in host health against *L. garvieae* [191]. The rising incidence of *L. garvieae* and related species like *L. petauri* in freshwater and marine farms, including recent European outbreaks beyond traditional hosts, underscores the urgent need for effective control methods. Current reliance on biosecurity, vaccination, and antibiotics is challenged by resistance and environmental concerns, positioning phage therapy as a promising alternative [192].

### 9.4. Phage Control of Pseudomonas proteus

*Pseudomonas proteus* is a Gram-negative bacterium from the genus *Pseudomonas* and is a causative agent of bacterial hemorrhagic septicemia in sweetfish (*Plecoglossus altivelis*), leading to considerable economic losses in aquaculture [193]. This disease affects sweetfish at all growth stages and is marked by an acute onset and high mortality. Challenge tests have revealed the bacterium’s strong infectivity, with a median lethal dose (LD_50_) as low as 10^1.2^ CFU per fish, underscoring its virulence [194]. In a key study, two specific lytic bacteriophages targeting *P. proteus* were isolated from diseased sweetfish tissues and aquaculture pond water, indicating potential for phage therapy in managing this pathogen. The first phage, designated PPpW-3, was morphologically classified within the *Myoviridae* family and produced small plaques on bacterial lawns. The second, PPpW-4, belonged to the *Podoviridae* family (historically referred to as *Brachyviridae*) and formed larger plaques [193].

### 9.5. Phage Control of Vibrio harveyi

*Vibrio harveyi* is a Gram-negative bacterium commonly found in seawater and the intestines of marine animals, including shrimp. It is a major pathogen in shrimp hatcheries and culture ponds, causing luminous vibriosis and substantial economic losses [195]. Vinod et al. isolated a *V. harveyi*-specific lytic phage from a shrimp pond on India’s west coast, morphologically classified as a member of the *Longicaudophage* family. In controlled experiments, shrimp challenged with *V. harveyi* (10^5^ CFU/mL) showed significantly improved survival rates when treated with phage cocktail (10^9^ PFU/mL). Immediate phage treatment resulted in 80% survival, while a second dose after 24 h yielded 70% survival, compared to only 25% in untreated controls [195]. Scaling up, a pilot trial in commercial shrimp farms using 5000 L tanks with 35,000 shrimp each, maintained a phage titer of 2 × 10^8^ PFU/L. The phage-treated group achieved an 86% survival rate, outperforming the antibiotic-treated group (40%) and untreated controls (17%) [195]. Similarly, Karunasagar et al. (2007) [196] identified four broad-host-range phages against *V. harveyi*, with Viha8 and Viha10 lysing approximately 70% of the tested strains. Their pilot study demonstrated survival rates of 86–88% in phage-treated groups, significantly higher than oxytetracycline (68%) and kanamycin (65%) controls [196].

Under controlled laboratory conditions, phage treatments have been shown to reduce bacterial burden and improve animal survival in several aquaculture models; in select studies, phage cocktails performed comparably to or exceeded antibiotic controls. However, these results should be interpreted cautiously, as outcomes vary considerably across host species, pathogen strains, phage doses, infection models, and experimental conditions. No generalized superiority of phage therapy over antibiotics can be assumed without context-specific evaluation. Further studies by Crothers-Stomps et al. (2010) isolated over ten *V. harveyi*-specific lytic phages exhibiting strong in vitro lytic activity [197]. The researcher isolated three phages (VHM1, VHM2, VHS1) and showed that phage application at 10^9^ PFU/mL significantly improved shrimp survival [198]. Another study demonstrated the efficacy of two phages, vB_VhaS-a and vB_VhaS-tm, in protecting green-striped abalone from *V. harveyi* infection, increasing survival to 70% compared to 0% in untreated groups [199].

Recent advances include the development of phage cocktails that target multiple *Vibrio* species, enhancing therapeutic efficacy and reducing bacterial resistance. A nucleus-forming vibriophage cocktail reduced shrimp mortality by over 90% in *Vibrio parahaemolyticus* infections [200]. Broad-host-range phages such as vB_VhaS-R18L show promise due to their environmental stability and wide lytic spectrum [201]. Additionally, phage cocktails composed of vB_Vc_SrVc2 and vB_Vc_SrVc9 selectively eliminated *Vibrio* species in shrimp postlarvae, improving health and reducing pathogen load [202].

Although the above findings derive from shrimp, abalone, and oyster models, several principles are mechanistically transferable to sea cucumber aquaculture. First, phage cocktail strategies that target multiple *Vibrio* species are directly applicable to *A. japonicus* farming, where *V. splendidus*, *V. alginolyticus*, and *V. harveyi* frequently co-occur. Second, the demonstrated efficacy of feed-based delivery in shrimp provides a technical basis for similar approaches in sea cucumber, given the filter-feeding and sediment-grazing behavior of *A. japonicus*. However, direct extrapolation should be made cautiously: the immunological architecture of sea cucumbers (invertebrate, coelomocyte-based immunity) differs substantially from fish and shrimp, and phage survival in the unique chemical and microbial environment of sea cucumber culture ponds requires species-specific validation before therapeutic protocols from other aquaculture systems can be adopted.

### 9.6. Phage Control of Vibrio parahaemolyticus

*Vibrio parahaemolyticus* is a Gram-negative bacterium commonly found in coastal sediments and seafood such as fish, shrimp, and shellfish. It is a major pathogen in marine aquaculture, causing diseases in fish, clams, oysters, and prawns, leading to significant economic losses [203,204]. Additionally, *V. parahaemolyticus* is a recognized foodborne pathogen in humans, causing symptoms like vomiting, diarrhea, abdominal pain, and watery stools, raising serious food safety concerns [205]. Researchers isolated and characterized a lytic bacteriophage, VPp1, specific to *V. parahaemolyticus*, which exhibited a rapid incubation period of only 10 min, indicating potential for swift bacterial control in aquaculture disease management [206].

Further studies by Rong et al. (2014) [207] assessed the protective efficacy of phage VPp1 against *V. parahaemolyticus* infections in oysters under varying multiplicities of infection (MOI = 10, 1, and 0.1) and temperatures (12 °C, 16 °C, 20 °C, 22 °C). The optimal protection was observed at 16 °C with an MOI of 0.1, reducing *V. parahaemolyticus* by 2.35 to 2.76 log CFU/g in oyster tissues. Another investigation evaluated phage cocktails comprising three lytic phages (VP-1, VP-2, and VP-3), comparing single versus combined treatments [207]. Phage mixtures significantly improved bacterial lysis compared to single phages VP-1 or VP-2, but the potent lytic activity of VP-3 alone was comparable to the cocktail’s effect, suggesting that highly effective single phages may sometimes replace multi-phage formulations [157,208].

## 10. Prospects of Phage Application in Aquaculture

The future of phage application in aquaculture depends on improved phage formulation, better delivery systems, and more field-based validation under commercial conditions. Particular attention should be given to host-range limitations, resistance development, storage stability, and regulatory approval. For sea cucumber farming, phage therapy is most likely to succeed as part of an integrated disease-management strategy rather than as a stand-alone intervention:

1. For *A. japonicus* aquaculture, the host specificity of phages allows targeted control of pathogenic Vibrio species without disrupting the benthic microbial communities that support the sea cucumber’s filter-feeding and nutrient cycling. Field studies should monitor shifts in sediment microbiota following phage application to ensure ecological stability.
2. Feed-based phage delivery is the most practical route for grow-out systems, but phage stability in pelleted or dried feeds must be validated. Encapsulation technologies, such as alginate beads or microparticle formulations, have shown promise in preserving phage viability through feed processing and gastrointestinal transit in other aquaculture species and warrant testing in *A. japonicus.*
3. Phage self-replication within the water column may maintain therapeutic titers in immersion treatments, but the high organic load and microbial diversity of sea cucumber pond environments can reduce effective phage concentrations rapidly. Dosing strategies should account for phage adsorption to particulate matter and temperature-dependent inactivation.
4. Nursery systems, where juvenile *A. japonicus* are most vulnerable to *Vibrio*-associated skin ulceration, represent the highest-priority application site for phage therapy. Controlled nursery trials with validated phage cocktails targeting dominant local strains should be prioritized over grow-out trials, given the greater ease of dose control and the higher economic value per animal at this stage.
5. Phage performance in aquaculture is strongly influenced by environmental conditions, including temperature, pH, salinity, and organic load, which can alter phage stability and host adsorption efficiency. Results obtained under controlled laboratory conditions may not translate directly to field settings, where high biomass, variable water quality, and fluctuating pathogen loads can reduce phage performance.
6. Phages are environmentally safe, biodegradable, and leave no toxic residues. Unlike antibiotics, phages do not pose risks of accumulating harmful substances in the aquatic environment, making them a sustainable choice for managing bacterial diseases in aquaculture. Phage therapy offers targeted control of bacterial pathogens in aquaculture. Beyond pathogen removal, phage application may influence microbial community composition, ecological balance, and functional stability in aquaculture systems, with potential consequences for both disease control and ecosystem resilience. Although many studies report favorable outcomes, the evidence base remains heterogeneous, and comparisons across studies should account for differences in host species, pathogen strain, delivery route, phage dose, and environmental conditions. Beyond direct antibacterial therapy, phage platforms may be explored for antigen display and vaccine development in aquatic disease control.

## 11. Limitations of Phage Therapy

Despite the promise of phage therapy for sea cucumber aquaculture, several limitations remain to be addressed before broad-field application. The narrow host range of many phages means that a single phage may not be effective against all circulating bacterial strains, necessitating phage cocktails or strain-specific selection. Regulatory approval for phage-based products remains challenging because safety evaluation, manufacturing standards, and environmental release requirements differ across countries and regions. In addition, bacterial resistance to phages may emerge during repeated use, reducing long-term treatment efficacy and necessitating continuous monitoring of phage-host interactions [9]. Phage therapy has shown promising efficacy against aquaculture pathogens. Bacterial resistance to phages may emerge through receptor alteration, restriction-modification systems, abortive infection, or CRISPR-Cas defense, potentially reducing long-term therapeutic efficacy. Environmental conditions such as temperature, pH, salinity, and storage stability may also affect phage survival and activity in aquatic systems, potentially limiting performance under farm conditions. Delivery efficiency is another concern because oral, immersion, and injection routes differ in stability, uptake, and practical feasibility in large-scale aquaculture. Finally, regulatory approval, quality control, and standardization of phage preparations remain important barriers to practical implementation [209].

## 12. Conclusions

Phage therapy represents a promising and sustainable strategy for controlling bacterial diseases in sea cucumber aquaculture. The available evidence shows that phages can reduce pathogen load, improve survival, and support a move away from antibiotic dependence. However, broader adoption will require stronger field validation, better formulation, more standardized production, and clearer regulatory pathways. With continued research, phage therapy may become an important tool for safer and more sustainable sea cucumber farming.

## Figures and Tables

**Table 4:** Phage applications targeting key aquaculture pathogens.

Aquaculture Species	Disease Name	Pathogen	Phage (s)	Phage Source	References
Japanese Eel (*Anguilla Japonica*)	Edwardsiellosis	*Edwardsiella tarda*	ET-1	Aquaculture pond water	[170]
Yellowtail Amberjack (*Seriola quinqueradiata*)	Lactococcosis	*Lactococcus garvieae*	Long-tail phages PLgY-16, PLgY-30, PLgW-1	Natural seawater and diseased fish wastewater	[171]
Giant Tiger Prawn (*Penaeus monodon*)	Vibriosis	*Vibrio harveyi*	Myovirus VHLM	Aquaculture wastewater	[172]
*Sweetfish* (*Plecoglossus altivelis*)	Hemorrhagic ascites disease	*Pseudomonas plecoglossicida*	Phage cocktail PPpW-4, PPpW-3	Diseased fish and aquaculture wastewater	[173]
Brook Trout (*Salvelinus fontinalis*)	Furunculosis	*Aeromonas salmonicida* HER1107	Phage HER110	Aquaculture wastewater	[174]
Giant Tiger Prawn (*Penaeus monodon*)	Vibriosis	*Vibrio harveyi*	Long-tail phage	Shrimp farms, west coast of India	[175]
Giant Tiger Prawn (*Penaeus monodon*)	Vibriosis	*Vibrio harveyi*	Multiple long-tail phages	Oyster tissues and shrimp farm water	[176]
Atlantic Salmon (*Salmo salar*)	Furunculosis	*Aeromonas salmonicida* 78027	Phage cocktail O, R, B		[177]
Olive Flounder (*Paralichthys olivaceus*)	Streptococcosis	*Streptococcus iniae*	Multiple phages	Fishpond water	[178]
Pacific White Shrimp (*Penaeus Vanmamei*)	Vibriosis	*Vibrio harveyi*	6 long-tail and 1 myovirus phage cocktail	Shrimp farms and hatchery waters, India	[179]
Catfish (*Clarias batrachus*)	Columnaris disease	*Flavobacterium columnare*	9 phages FCP1 to FCP9	Aquaculture water and sediment	[180]
Channel Catfish (*Ictalurus punctatus*)	Enteric septicemia	*Edwardsiella ictaluri*	Phages eiDWF, eiAU, eiMSLS	Aquaculture water	[181]
*Plecoglossus altivelis*	BCWD	*Flavobacterium psychrophilum*	PFpW-3, PFpC-Y, PFpW-6, PFpW-7, PFpW-8	Ayu breeding pond water	[182]
Oyster (*Crassostrea gigas*)	Bacterial disease	*Vibrio parahaemolyticus*	Phage VPp1	Aquatic market water	[183]
Japanese Sea Cucumber (*Apostichopus japonicus*)	Skin ulcer disease	*Vibrio alginolyticus*	Phage PVA1	Aquaculture wastewater and sediment	[184]

## Data Availability

No new data were created or analyzed in this study. Data sharing does not apply to this article, as it is based on previously published literature and publicly available sources.

## Supplementary Materials

The following supporting information can be downloaded at: https://www.mdpi.com/article/10.3390/life16060989/s1, Table S1: Current classification of Caudovirales by ICTV 2023.
