# Supplementary material for: Phage Therapy for Sustainable Sea Cucumber Aquaculture
Source: Life (Basel). 2026 Jun 11;16(6):989. doi: 10.3390/life16060989 (PMC13301823; doi:10.3390/life16060989)
Supplement: Supplementary file 1 [file life-16-00989-s001.zip › life-4260762-supplementary.pdf]

TABLE S1 Current classification of *Caudovirales* by ICTV 2023.

Historical Family	Subfamily	Genus	Representative Species
Ackermannviridae	***Aglimvirinae***	*Ag3virus*	Salmonella phage Ag3; Salmonella phage SKML39
		Limestonevirus	Soft rot bacteriophage RC2014; Limestone phage
	Cvivirinae	*Cba120virus*	Escherichia phage CBA120; Escherichia phage PhaxI; Salmonella phage 38; Salmonella phage Det7; Salmonella phage GG32; Salmonella phage SFP10
		Vi1virus	Escherichia phage ECML4; Salmonella phage SJ2; Salmonella phage STML131; Salmonella phage ViI
Myoviridae	***Eucampyvirinae***	*Cp220virus*	Campylobacter phage CP220
		Cp8virus	Campylobacter phage CP81
	Peduovirinae	*Hp1virus*	Haemophilus phage HP1
		P2virus	Escherichia coli phage P2
	Spounavirinae	*Kayvirus*	Staphylococcal phage K
		P100virus	Listeria phage P100
		Silviavirus	Staphylococcal phage Remus
		Spo1virus	Bacillus subtilis phage SPO1
		Twortvirus	Staphylococcal phage Twort
	Tevenvirinae	*Cc31virus*	Enterobacteriaceae phage CC31
		Js98virus	Escherichia coli phage JS98
		Rb49virus	Escherichia phage RB49
		Rb69virus	Escherichia phage RB69
		S16virus	Salmonella phage S16
		Schizot4virus	Vibrio phage KVP40
		Sp18virus	Shigella phage SP18
		T4virus	Escherichia coli phage T4
	Vequintavirinae	*Cr3virus*	Enterobacter sakazakii phage CR3
		Se1virus	Salmonella phage SE1
		V5virus	Escherichia coli phage V5
		Agatevirus	Bacillus phage Agate
		Ap22virus	Acinetobacter phage AP22
		B4virus	Bacillus phage B4
		Bastillevirus	Bacillus phage Bastille
		Bc431virus	Bacillus phage Bc431
		Bcep78virus	Burkholderia phage Bcep781
		Bcepmuvirus	Burkholderia phage BcepMu
		Biquartavirus	Aeromonas phage 44RR2
		Bxz1virus	Mycobacterium phage I3
		Cd119virus	Fusobacterium phage phiCD119
		Cp51virus	Bacillus phage CP51
		Cvm10virus	Escherichia coli phage CVM10

		Felixo1virus	Salmonella phage FelixO1
		Hapunavirus	Halomonas phage HAP1
		Kpp10virus	Pseudomonas phage KPP10
		Muvirus	Escherichia coli phage Mu
		Myohalovirus	Halophilic phage phiH
		Nit1virus	Bacillus phage NIT1
		P1virus	Escherichia coli phage P1
		Pakpunavirus	Pseudomonas aeruginosa phage PAKP1
		Pbunavirus	Pseudomonas aeruginosa phage PB1
		Phikzvirus	Pseudomonas aeruginosa phage phiKZ
		Rheph4virus	Rhizobium phage RHEph4
		Secunda5virus	Aeromonas phage 25
		Tg1virus	Yersinia pestis phage TG1
		Vhmlvirus	Vibrio phage VHML
		Vi1virus	Salmonella phage ViI
		Wphvirus	Bacillus phage WPh
Podoviridae	***Autographivirinae***	*Kp34virus*	Klebsiella phage KP34
		Phikmvvirus	Pseudomonas aeruginosa phage phiKMV
		Sp6virus	Salmonella phage SP6
		T7virus	Escherichia coli phage T7
	Picovirinae	*P68virus*	Staphylococcus phage 44AHJD
		Phi29virus	Bacillus phage phi29
		Bcep22likevirus	Burkholderia phage Bcep22
		Bpp1virus	Bordetella pertussis phage BPP1
		Cba41virus	Cytophaga phage Cba41
		Epsilon15virus	Salmonella phage Epsilon15
		F116virus	Pseudomonas aeruginosa phage F116
		G7cvirus	Escherichia coli phage G7C
		Lit1virus	Pseudomonas aeruginosa phage LIT1
		Luz24virus	Pseudomonas aeruginosa phage LUZ24
		N4virus	Escherichia coli phage N4
		Nonanavirus	Salmonella phage 9NA
		P22virus	Salmonella phage P22
		Pagevirus	Bacillus phage Page
		Phieco32virus	Escherichia coli phage phiEco32
		Vp5virus	Vibrio phage VP5
Siphoviridae	***Guernseyvirinae***	*Jerseyvirus*	Salmonella phage Jersey
		K1gvirus	Escherichia coli phage K1G
		Sp31virus	Salmonella phage SP31
	Tunavirinae	*Kp36virus*	Klebsiella phage KP36
		Rogue1virus	Escherichia coli phage Rogue1
		Rtpvirus	Escherichia coli phage Rtp
		T1virus	Escherichia coli phage T1
		Tlsvirus	Escherichia coli phage TLS
		Andromedavirus	Bacillus phage Andromeda
		Barnyardvirus	Mycobacterial phage Barnyard

	Bignuzvirus	Mycobacterial phage Bignuz
	Biseptimavirus	Staphylococcal phage 77
	Bronvirus	Mycobacterial phage Bron
	C2virus	Lactococcal phage c2
	C5virus	Lactobacillus phage c5
	Cba181virus	Cellulophaga phage Cba181
	Cbastvirus	Cellulophage phage ST
